# Artificial light and nocturnal activity in gammarids

**DOI:** 10.7717/peerj.279

**Published:** 2014-03-06

**Authors:** Elizabeth K. Perkin, Franz Hölker, Stefan Heller, Rüdiger Berghahn

**Affiliations:** 1 Leibniz Institute of Freshwater Ecology and Inland Fisheries, Berlin, Germany; 2 Institute of Biology, Freie Universität Berlin, Berlin, Germany; 3 Umweltbundesamt, Berlin, Germany

**Keywords:** Acclimation, Gammarus, Invertebrate drift, Light pollution, Multispecies freshwater biomonitor

## Abstract

Artificial light is gaining attention as a potential stressor to aquatic ecosystems. Artificial lights located near streams increase light levels experienced by stream invertebrates and we hypothesized light would depress night drift rates. We also hypothesized that the effect of light on drift rates would decrease over time as the invertebrates acclimated to the new light level over the course of one month’s exposure. These hypotheses were tested by placing *Gammarus* spp. in eight, 75 m × 1 m artificial flumes. One flume was exposed to strong (416 lx) artificial light at night. This strong light created a gradient between 4.19 and 0.04 lx over the neighboring six artificial flumes, while a control flume was completely covered with black plastic at night. Night-time light measurements taken in the Berlin area confirm that half the flumes were at light levels experienced by urban aquatic invertebrates. Surprisingly, no light treatment affected gammarid drift rates. In contrast, physical activity measurements of *in situ* individually caged *G. roeseli* showed they increased short-term activity levels in nights of complete darkness and decreased activity levels in brightly lit flumes. Both nocturnal and diurnal drift increased, and day drift rates were unexpectadly higher than nocturnal drift.

## Introduction

Light pollution is an increasing problem across the globe (Longcore & Rich, 2004; Hölker et al., 2010). Based on satellite data from 1996–1997, 71% of the population of the United States, and 51% of the population of the European Union can no longer see the Milky Way, even under the best conditions (Cinzano, Falchi & Elvidge, 2001) due to ambient light from artificial sources. Although artificial lights are widespread, their potential effects on freshwater biotic communities have received relatively little attention to date (Longcore & Rich, 2004; Perkin et al., 2011). Additionally, increases in “sky glow”, or the general increase in light emitted from large urban areas, can influence peri-urban and even rural areas (Perkin et al., 2011). Aquatic bodies located near (within 10–20 km) urban areas could be exposed to light levels close to, or equal to that of a full moon (Moore et al., 2000; Kyba et al., 2011). Clear, small streams are most likely to be affected by artificial lights at night, because they are most likely to transmit light through from the surface to the benthos (Moore, Kohler & Cheers, 2006). This change in the environment experienced by aquatic organisms at night could have unintended consequences for their behavior, particularly behaviors that are triggered by changes in light availability.

The majority of stream invertebrates drift during dark hours of the night, most likely to avoid predation by visual predators such as drift-feeding fish (Flecker, 1992). While drift-feeding fish may still catch prey during the night, their efficiency is reduced by diminished visual acuity (Fraser & Metcalfe, 1997). Artificial light at night may allow visually foraging fish to capture more prey, provided that drifting invertebrate prey is still available.

It is generally understood that the changes in drift behavior are exogenously controlled by changes in ambient light levels, and do not result from endogenous circadian rhythms (Bishop, 1969). Bishop (1969) showed that even brief exposure to light in the middle of the night greatly decreased drift; similarly, exposure to darkness in the middle of the day resulted in an increase in drift. It is unclear, however, if invertebrates will continue to not drift if constantly exposed to low light levels, or if they eventually resume drifting despite the light, due to increased competitive interactions. For instance, invertebrates may have higher drift rates when population density is high (Walton Jr, Reice & Andrews, 1977), or when food availability is low (Hughes, 1970; Hershey et al., 1993).

We conducted this study in Berlin (Germany) during late fall/early winter of 2009 when nighttime is roughly twice as long as daylight. We used *Gammarus* spp. as a study species because they have a high propensity to enter the drift, and therefore be available to predaceous fish (Rader, 1997), and they are an abundant shredder taxa in the sand-bottom lowland streams that are common in Northern Germany. By placing *Gammarus* spp. in eight, 75 m × 1 m artificial flumes for four weeks, we wanted to determine if artificial light would alter downstream drift in gammarids resulting in changed availability as fish prey and distribution patterns. We hypothesized that the long duration of the night in the winter study period would lead to artificial light having an even more pronounced effect on the drift rates of aquatic invertebrates. To provide a better understanding of the short-term response of *Gammarus roeseli, G. pulex* and *Dikerogammarus villosus* to artificial light at night we conducted a pilot study in which we ran small-scale experiments looking at their immediate individual behavioral response to different levels of artificial light. Our study objectives were to determine (1) if nighttime invertebrate activity decreases during short-term exposure to artificial light, and (2) if the decrease in drift remains constant over time, or lessens with acclimation to the new light environment. Experimental light levels were compared to those measured in the field in the Berlin area allowed us to know that the results of the drift experiment were from ecologically relevant light levels.

## Materials and Methods

The experiment was run using the indoor stream mesocosms of the artificial pond and stream system (FSA) at the test area of the German Federal Environment Agency (Umweltbundesamt, UBA) in Marienfelde (Berlin). The flumes were housed in a large warehouse-type building and contains eight indoor flumes that are each 75 m long and are constructed from green fiberglass reinforced polyester (Berghahn et al., 1999). The building kept flumes warm enough to prevent freezing, but did not prevent seasonal temperature differences. Moonlight entering through skylights in the building was negligible, as the roof was covered in snow throughout most of the experiment. Flume width is generally 1 m, except in four pool locations of 3 m length in each channel that are 1.2 m wide (Mohr et al., 2005). The pool sections were planted with the macrophyte species *Sparganium erectum (L.)* to provide features similar to those encountered in the field, such as water turbulence and hiding places for aquatic animals. The substrate in all flumes was washed, uncontaminated sand (0–2 mm diameter) from a gravel pit that had been covered with a thin layer of uncontaminated fine sediment from a lake (Schmachter See, Mecklenburg Western Pommerania, Germany). The water depth in all flumes was 0.2 m. The flumes were operated in a circular flow mode by integrated screw pumps at a flow rate of 0.10 m s^−1^. Flow rate, turbidity, dissolved oxygen concentration, pH, conductivity, and water temperature in each flume were taken automatically every hour throughout the duration of the experiment. For details about the measurement equipment see Mohr et al. (2005).

### Invertebrate collection

Organisms were collected in the fall from the River Spree near the small town of Mönchwinkel, the Demnitzer Mühlenfließ, and the Löcknitz near the small town of Kienbaum, approximately 38 km, 50 km, and 23 km southeast from the city center of Berlin, Germany. The streams in these areas are quite dark and exposure to artificial light before the experiment was minimal. During the winter, the majority of invertebrates found in all of these streams are *Gammarus* spp. Sampling, transport, and stocking followed the method described by Mohr et al. (2012). Accordingly, 85 mesh sacks (mesh opening 6 × 6 mm, stretched mesh), each filled with 100 g of organic triticale straw were left in the Spree from 30 September to 7 October. After collection, straw sacks were immediately taken to the flumes. Nine sacks were placed in each of the eight flumes and opened so that the straw and animals were distributed over the bottom of the flume. Prior to stocking, several “ripples” were artificially created in the sand substrate in order to capture the straw from the sacks. The ripples were at a right angle to the flow direction and were ∼8 cm wide and 1 cm deep. Thus, three habitats (walls, sand areas, straw areas) were available in each flume. Of the sacks collected in the field, five were set aside to determine the number and species of invertebrates collected. From these five sacks it was determined that not enough invertebrates were present to conduct the experiment, so two further collections were made; one at the Demnitzer Mühlenfließ (15–26 October) and at the Löcknitz (6–13 November).

**Figure 1:**
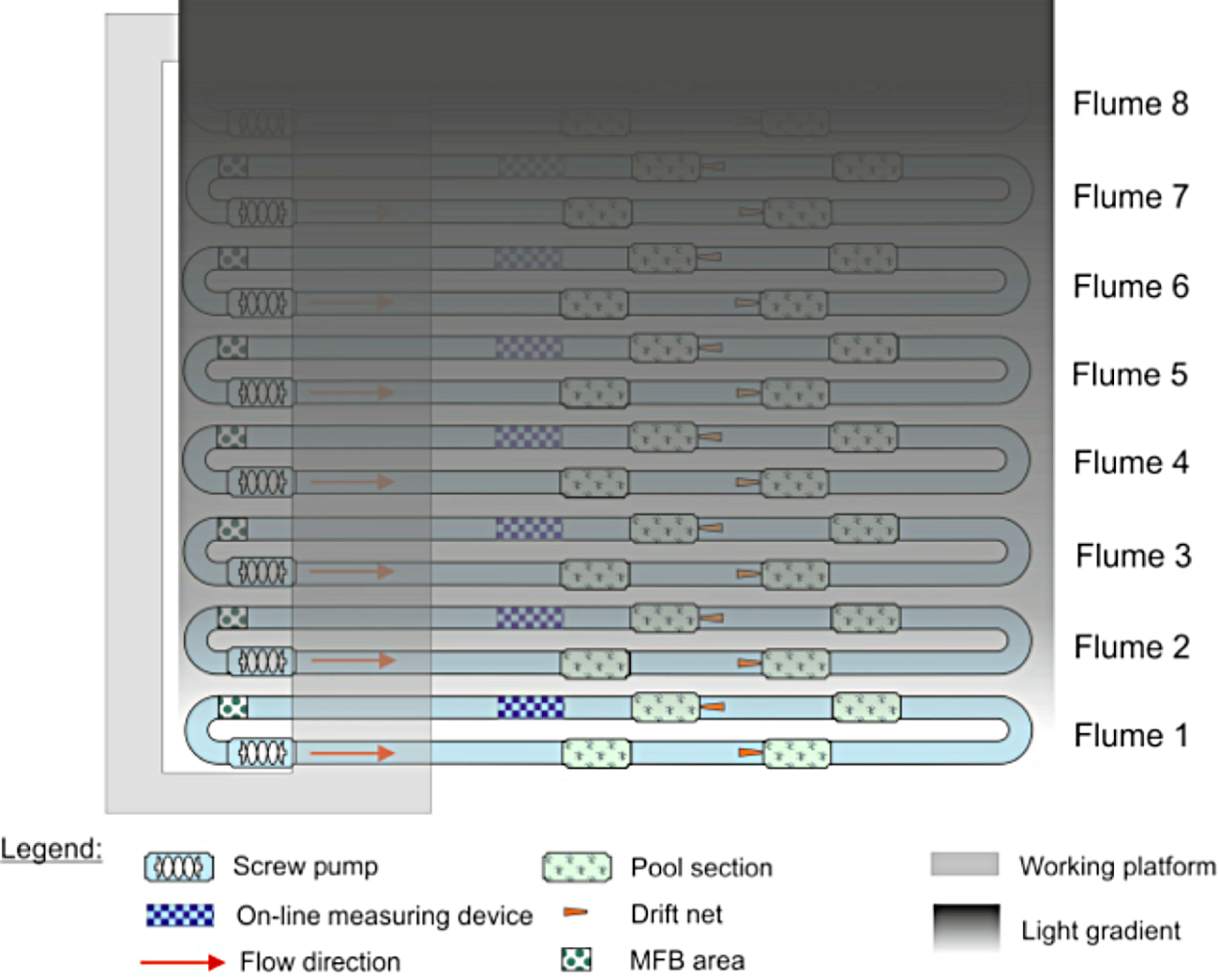
Configuration of the eight indoor flumes and the light gradient. All flumes were exposed to natural light during the day. At night, one bank of lights was left on over flume 1, creating a gradient of light over the other seven flumes. Flume 8 was covered with black plastic to serve as a control.

Because only eight flumes were available and we were interested in testing the effects of multiple light levels on invertebrate drift, we decided to have a gradient of light over the flumes rather than a replicated design with only one light level. Illuminating one stream at the far end of the experimental hall created a decreasing gradient of light over the other streams in the facility (Fig. 1). To create complete darkness, the stream most distant from the fully illuminated one was covered with black light-tight foil every night from 1600–0800. Artificial light was provided by 23, Osram Biolux T8 L 58W/965 G13, 6500 K. Light levels were measured twice during the experiment, once at roughly the mid-point (30 November) on the night before a full moon, and the other at the end of the experiment (14 December) on the night before a new moon. Both measurements were made with an ILT1700 light meter (range: 0.00167–1,670,000 lx, International Light Technologies, Peabody, MA), and were taken between 1730 and 1800 by placing the light meter on the surface of the stream substrate. The artificial light spectrum was measured with an OceanOptics Spectrasuite^®^ (Dunedin, FL).

### Drift experiment

Animals were allowed to acclimate to the system for at least one week before artificial night lighting of the system began on 20 November and ended on 15 December. Invertebrate drift was sampled with two drift nets (dimensions: mouth opening = 15 × 7.5 cm, length = 140 cm, mesh = 283 µm), which were placed downstream of riffles two and four in the middle of each stream just above the sediment surface (Fig. 1). Drift samples were collected both during the day (0800–1600, on the 19 November) and during the night (1600–0800, on the 20 November), prior to the initiation of artificial night light and were used to determine the drift in the flumes under relatively natural conditions.

Two drift samples from each stream were taken during the day (from 0800–1600) on the 23 and 30 November, and the 7 and 14 December. Another two drift samples were taken from each stream during the night (from 1600–0800) on the 24 November, and the 1, 8, and 15 December. Due to the low diversity of invertebrates in the system, we were able to immediately identify and count the organisms collected in the drift samples and then return them to their respective streams within one hour, where they were observed to behave normally upon their release. Because it was not possible to randomize treatments by stream, each flume was considered an experimental unit and replication was through time.

### Post-experiment benthic and periphyton sampling

To estimate the number of invertebrates per flume, benthic sub-samples were collected at the end of the experiment. Because each flume had three distinct habitats created by the presence or absence of straw and the walls, we took five sub-samples each from both straw and bare sediment areas in each flume by means of tube corers (inner diameter 18.7 cm, suction sampling) and from the walls with a kick-sampler modified to scrape the walls (opening 30 cm) employing stratified random sampling techniques. The five samples of each stratum were pooled for each flume and fixed in 80% ethanol for counting. Details on the sub-sampling protocol can be found in Mohr et al. (2012).

Periphyton growth during the experiment was measured with six sterile fiberglass reinforced polyester plates (gel-coated, 10 × 20 cm) that were placed upright in three flumes (at 416, 0.59 and 0.0 lx) at the beginning of the experiment. At the end of the experiment, all periphyton was scraped from the plates, diluted in 1 L water and filtered onto pre-weighed and dried Whatman GF/C 1.2 µm fiberglass filter. Periphyton on the filter paper was then dried overnight at 105°C and weighed.

### Multispecies freshwater biomonitor (MFB) experiment

Physical activity and drift behavior of apparently healthy caged adult *Dikerogammarus villosus*, *G. roeseli*, and *G. pulex* were measured in flumes one (416 lx), four (0.59 lx), and eight (0 lx) with a Multispecies Freshwater Biomonitor^®^ (MFB, Gerhardt et al., 1994) during the time period of the drift experiment. The MFB uses a quadropole impedance conversion technique to detect the movements of the invertebrates. As the invertebrate in the chamber moves, it alters the conductivity and the electrical field in an alternating current created by electrodes on opposite walls of the test chamber. These changes are detected by another non-current carrying pair of electrodes and can be directly linked to different kinds of behavior (LimCo International GmbH, 2013).

Animals used in the MFB experiment were acclimated in large tubs for at least 24 h to the light regime of the flume and then six specimens were individually transferred into six transparent acrylic glass tubes (length 50 cm, inner diameter 1.8 cm), which were positioned directly underneath the water surface parallel to the direction of the flow. At either end of the tubes were two MFB chambers (length 6 cm) that were open at one side to the acrylic glass tube. Mesh (opening 1 mm) covered the other end of the chambers, which prevented the gammarid individuals from leaving the system, but allowed for flow-through of flume water. By using the acrylic glass tubes with the MFB chambers, it was possible to monitor both the activity rate in mV and presence or absence of the invertebrates. Flow rate in the tubes was measured at about 0.05 m s^−1^. At this flow rate, both *G. roeseli* and *G. pulex* are known to react to the current (Vobis, 1973). Organisms were always transferred into the tubes at approx. 14:00, were given the chance to acclimate for 1 h, and then monitored for 48 h while their activity was recorded continuously at 10 min intervals. In some cases 5 min intervals were used for greater resolution when activity levels were high. The animals were not fed during the MFB trials and could freely move from one end of the tube to the other, including the two measuring chambers. When animals were active in the upstream chamber for the majority of the 48 h period, we assumed this was indicative of positive rheotaxis. If animals were active in the downstream chamber for the majority of the 48 h period, we assumed this was indicative of downstream drift. In addition, organisms could be in the transparent tube between the two measuring chambers. Because the measuring chambers are only able to detect the presence of an organism when it is active, we were unable to distinguish between an organism in the connecting tube and one resting (i.e., not moving) in one of the measuring chambers. Because there were only 12 chambers available, the measurements of organisms in different light levels had to take place at different times. The behavior of all three species was recorded under conditions of permanent bright light (416 lx) during the first week of the experiment, but measurements of behavior in completely dark nights (0.0 lx) was only possible for *G. roeseli* and *G. pulex* during the last week of the experiment. The only species which was tested in the MFB at all three light levels, including dimmed light at night (0.59 lx), was *G. roeseli.*

### Light measurements in Berlin

In order to know if light levels in the experiment represented light levels that might be encountered by stream invertebrates in an urban area, we took light measurements at several locations in waterways throughout Berlin. Light measurements were taken in lx with an ILT1700 light meter. All measurements were taken at least 15 min after evening civil twilight, 15 min before morning civil twilight, and 15 min after the setting or before the rising of a new or three-quarter moon, when background illumination was lowest. Measurement locations were selected from a light map created from low-elevation flights over Berlin and observations on the ground (Kuechly et al., 2012), and were chosen to select a variety of light environments, from very dark to very bright. Most measurements were taken at an underwater depth of approximately 50 cm, though one reading was taken at a depth of 40 cm.

### Analysis

Benthos sampling revealed that there were different numbers of animals in each flume, so the drift catches were standardized relative to the number of invertebrates in each flume. Furthermore, to account for any differences there might have been in the flumes other than light, the relative night drift was divided in half prior to comparing day and night drift as it sampled the drift for 16 h while the day drift only sampled 8 h of drift

\begin{eqnarray*} \displaystyle \text{Night:Day Drift Rate}=({d}_{i}\ast 100)/{N}_{i}&&\displaystyle \end{eqnarray*}

where *d_i_*, the ratio of night to day drift in flume *i*, and *N_i_*, the total number of invertebrates in flume *i*.

The catches of the two synchronously exposed drift nets from the same flume were checked for normal distribution and equality of variance and then compared with a paired *t*-test. To test if invertebrate drift increased over the course of the experiment, regression analyses for night:day drift rate vs. light level, and for night:day drift rate vs. temperature was done. Alpha for these tests was lowered to 0.017 after a Bonferroni correction for the number of tests (two). The relationship between both night and day drift rate and week of experiment was also tested. An ANCOVA was used to find if the resulting models for night and day drift were significantly different from one another. Comparisons between the time gammarids spent in the upstream vs. downstream chambers of the MFB were made with Mood’s median test and Wilcoxon sign-rank test. Alpha for these tests was 0.05. Analyses were conducted using Stateasy software 2007 (Lozan & Kausch, 2007), Microsoft Excel, and R (R Development Core Team, 2011).

## Results

**Figure 2:**
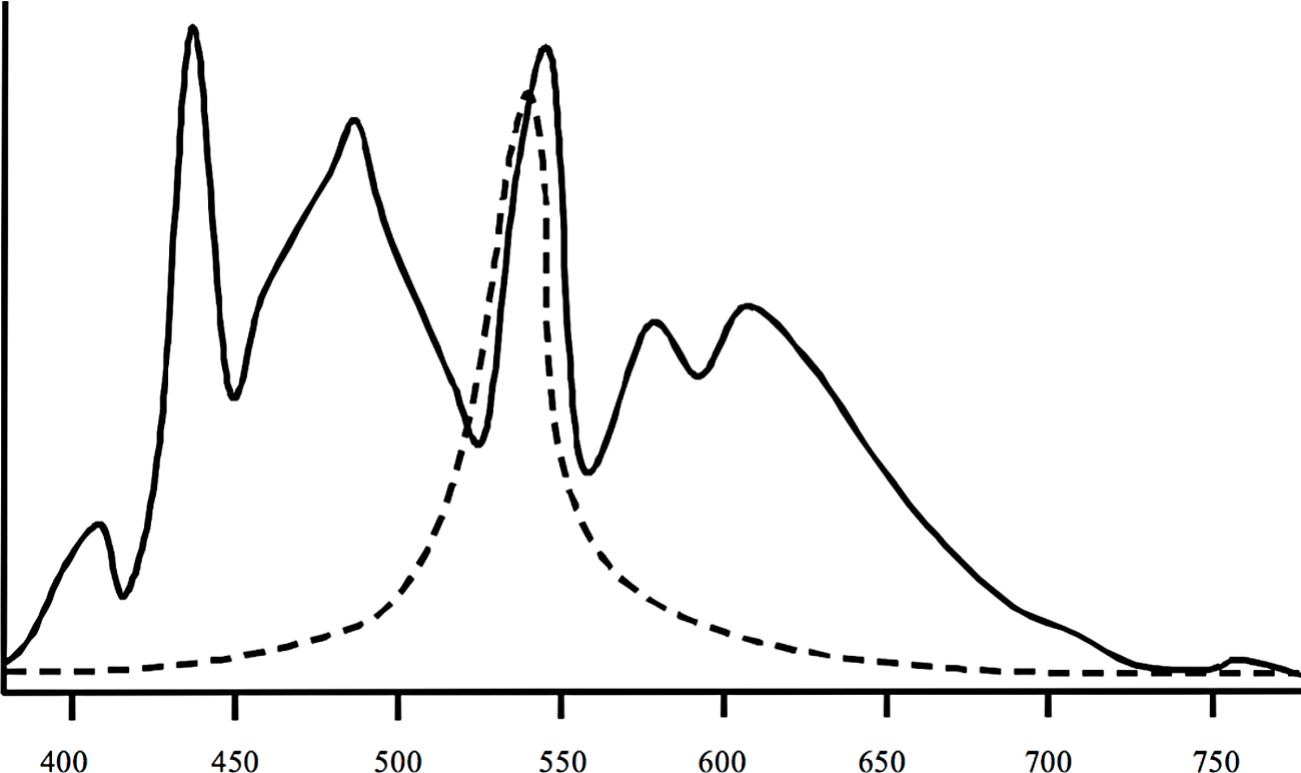
Spectral information of the lights (solid line) used in the study and of peak sensitivities of crustaceans (dashed line). The lights were Osram Biolx T8 L 58W/965 G13. Information on mysid crustaceans according to Porter et al. (2007) and Donner et al. (1994).

Spectral measurements (Fig. 2) of the artificial lights used in the experiment indicated a high degree of overlap between the artificial light spectrum and the peak light sensitivities of crustaceans (Donner et al., 1994; Porter et al., 2007). The water temperature in all flumes exhibited slight diurnal changes and decreased over the course of the experiment from ∼12.3 to 7.4°C. Mean water temperature was the same in all flumes (10.0°C, SD = 2.42°C). According to the water temperature on-line measurements in the flumes there was no spatial gradient with regard to their position in the hall. All on-line measurements of the other parameters were also almost identical between flumes. Water conductivity ranged from 490 to 530 µS cm^−1^, pH was between 8.1 and 8.3, and dissolved oxygen was 10.9 to 11.3 mg L^−1^. The water was very clear and the density of particles was ⩽ 1 ppm in all flumes.

### Drift experiment

The numbers of invertebrates caught in the two synchronously exposed drift nets in each flume were almost identical (*t* = 0.073, *p* > 0.05). For that reason, the mean of the two synchronous catches for each flume was used for further analysis.

**Figure 3:**
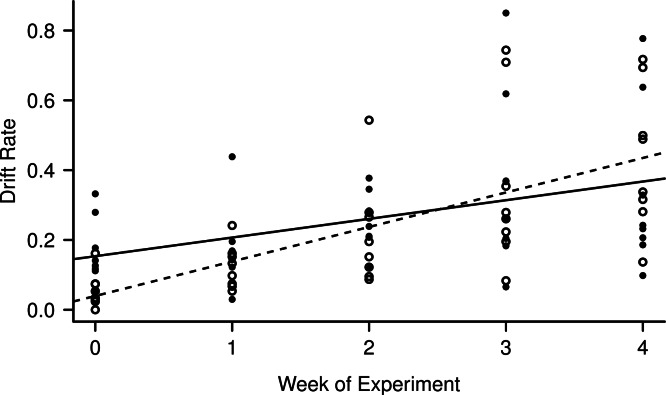
Increase in day (empty circles, dashed line) and night (filled circles, solid line) drift rates over the course of the experiment. The night catches were divided by two to match the time unit of the day catches (8 h) for comparison.

Light level and night:day drift were not linearly correlated (*R*^2^ = 0.007, *F*_1,38_ = 0.27, *p* = 0.61), while both day and night drift increased over the weeks of the experiment (Fig. 3) the day drift exceeded the night drift. The equation that best describes the increase in day drift rate over time is *Y* = 0.039 (Week) + 0.10 (*R*^2^ = 0.45, *F*_1,38_ = 32.59, *p* < 0.001), while the equation that best describes the increase in night drift over time is *Y* = 0.15 (Week) + 0.053 (*R*^2^ = 0.15, *F*_1,38_ = 7.89, *p* < 0.01). There was no significant difference between the increase in drift over time for day and night (*F*_1,76_ = 0.41, *p* = 0.53) and while there does appear to be an interaction between day and night drift, this is not significant (*F*_1,76_ = 3.11, *p* = 0.08). However, there was a significant linear correlation between night:day drift and temperature (*R*^2^ = 0.15, *F*_1,38_ = 7.89, *p* = 0.008).

### Benthic and periphyton biomass results

Benthic samples revealed that the density of invertebrates was variable between the flumes (mean = 3950 gammarids, SD = 2092.0), which is why we weighted the drift results with the corresponding population size. The highest number of invertebrates (8413 total, or 200 m^−2^) was in flume 5 (0.28 lx), while the lowest number (1882 total, or 45 m^−2^) was in flume 8 (0 lx).

Periphyton biomass was highest (172.2 mg m^−2^) in the brightest flume, but the lowest quantity grew under a medium light level in flume 4 (49.5 mg m^−2^) and an intermediate algal growth was in the dark flume 8 (82.9 mg m^−2^). Differences in periphyton biomass are also unlikely to be due to benthic densities of grazers, as flumes 1 and 4 had similarly high densities of grazers but the lowest and highest periphyton biomass, while flume 8 had the lowest density of grazers, but an intermediates level of periphyton biomass.

### MFB experiment

**Figure 4:**
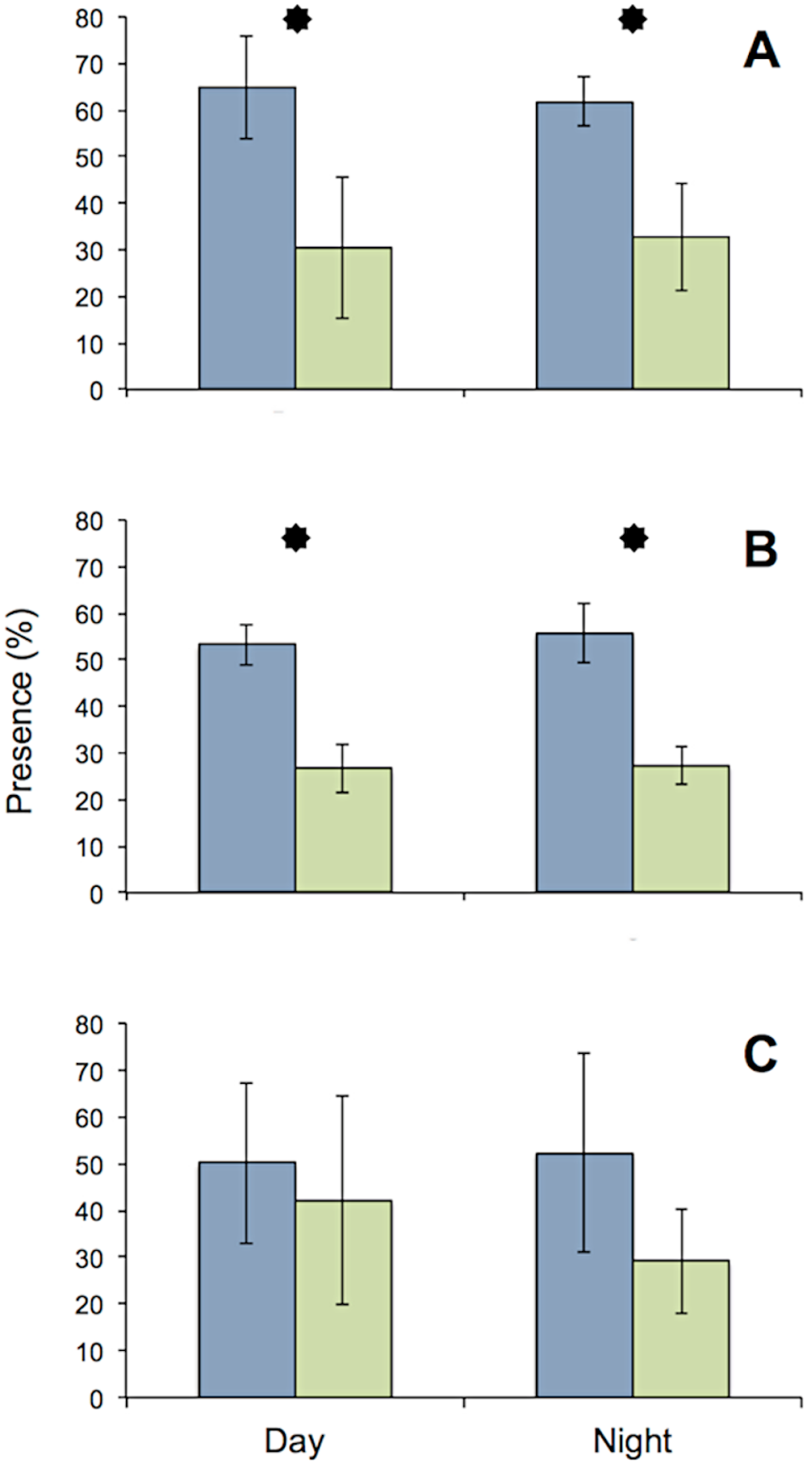
Comparison of presence in upstream (blue) and downstream (green) chambers of the MFB. Mean (± SE) presence (retention time) values of *G. roeseli* (A), *G. pulex* (B), and *D. villosus* (C) in the upstream and downstream chambers under permanently bright light (416 lx) during the day and night. Stars indicate significance between up and downstream chambers (Wilcoxon sign-rank test, level 5%).

All specimens used in the MFB behaved normally at the start and end of the experiment. The general activity pattern of all three species under bright light (416 lx) during both day and night was very similar, with the upstream chambers being visited more often than the downstream chamber. This finding was insignificant for *D. villosus*, because only three individuals from that species could be tested. Pronounced species-specific differences were evident when individuals (i.e., each test tube) were analyzed separately. Both *G. roeseli* and *G. pulex* frequently migrated from the upstream to the downstream test chamber and back again in the course of a day (Fig. 4) and were observed in the transparent connecting tube. In contrast, *D. villosus* moved between chambers only on rare occasions (i.e., after 1 or 2 days) and were present in the transparent connection tube only during quick migrations from one chamber to the other. Unlike the two *Gammarus* species, *D. villosus* seemed to be able to detect the presence of researchers. On the rare occasions when *D. villosus* was present in the connecting tube, *D. villosus* immediately sought shelter in one of the measuring chambers whenever someone approached the experimental setup. To exclude potential bias as a result of species-specific reactions, the experimental trials with *D. villosus* under bright light at night were not repeated and *D. villosus* was not used in further trials.

**Figure 5:**
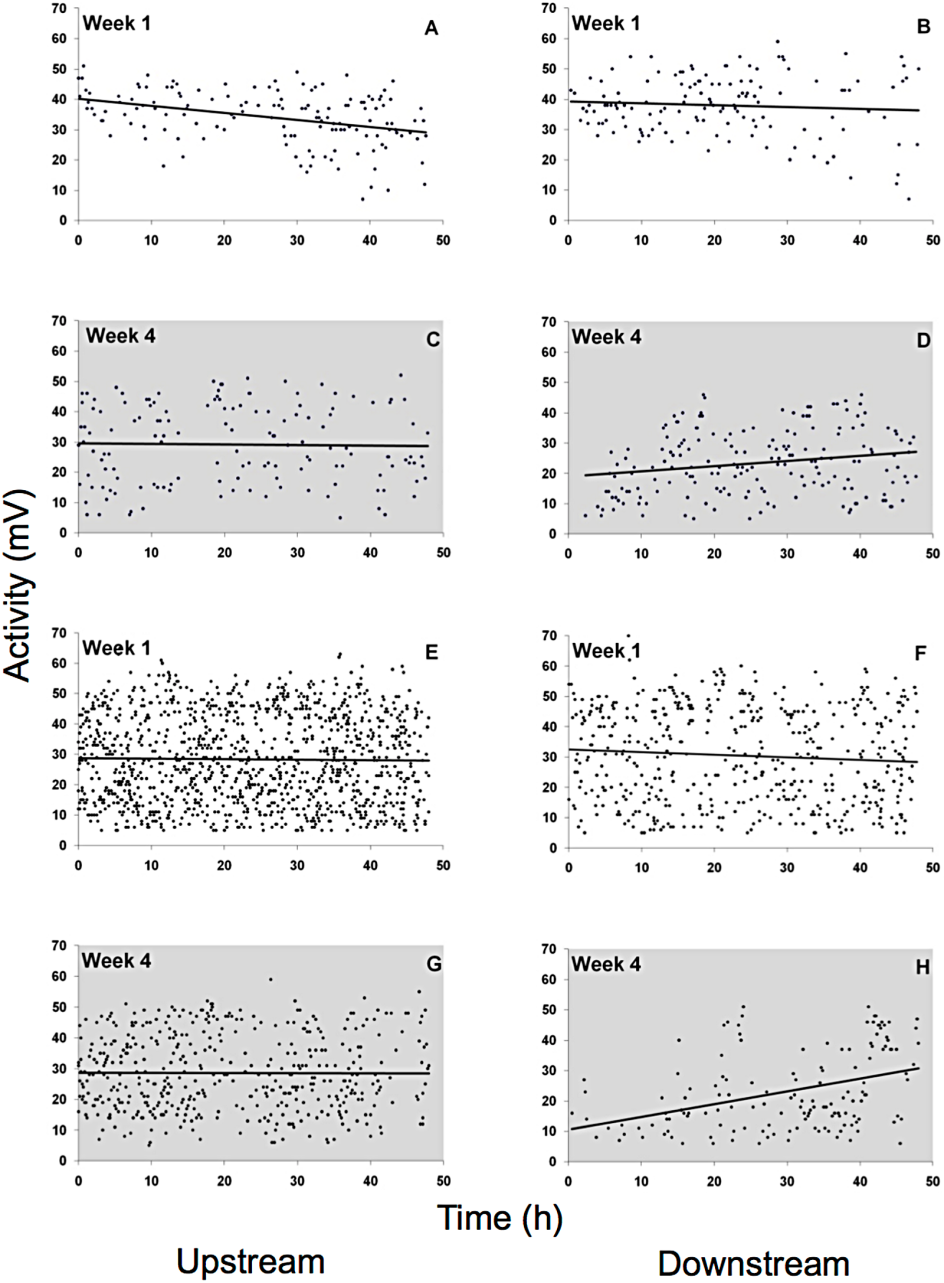
Physical activity of *G. roeseli* (A–D) and *G. pulex* (E–H). Activity was measured by changes in the electric field (mV) in the flow-through chambers of the Multispecies Freshwater Biomonitor (MFB). Each data point represents the mean of all synchronously tested specimens (see Materials and Methods). A, B, E, and F, light nights (416 lx), C, D, G, and H, dark nights (0 lx).

Spontaneous activity was high and highly variable in all gammarid species (Fig. 5, data for *D. villosus* not shown) and there were no pronounced diel activity changes. In the completely dark night treatment, activity increased in the downstream chamber in both *G. roeseli* (*R*^2^ = 0.04, *F*_3,9_ = 20.66, *p* = 0.05, increase by 42%, described by the equation *Y* = 0.17 (hour) + 19.04) and *G. pulex* (*R*^2^ = 0.15, *F*_3,9_ = 8.20, *p* = 0.05, increase by 282%, described by the equaiton *Y* = 0.42 (hour) + 10.49) over the course of the 48 h exposure. Under light at night conditions, however, physical activity (not drift) decreased significantly in both the upstream and downstream chamber for *G. roeseli* (13%, upstream: *Y* = −0.23 (hour) + 40.18, *R*^2^ = 0.14; downstream: *Y* = −0.06 (hour) + 39.29, *R*^2^ = 0.006) and *G. pulex* (27%, upstream: *Y* = −0.016 (hour) + 28.75, *R*^2^ = 0.0002; downstream: *Y* = −0.088 (hour) + 32.51, *R*^2^ = 0.006) (*p* < 0.05).

**Figure 6:**
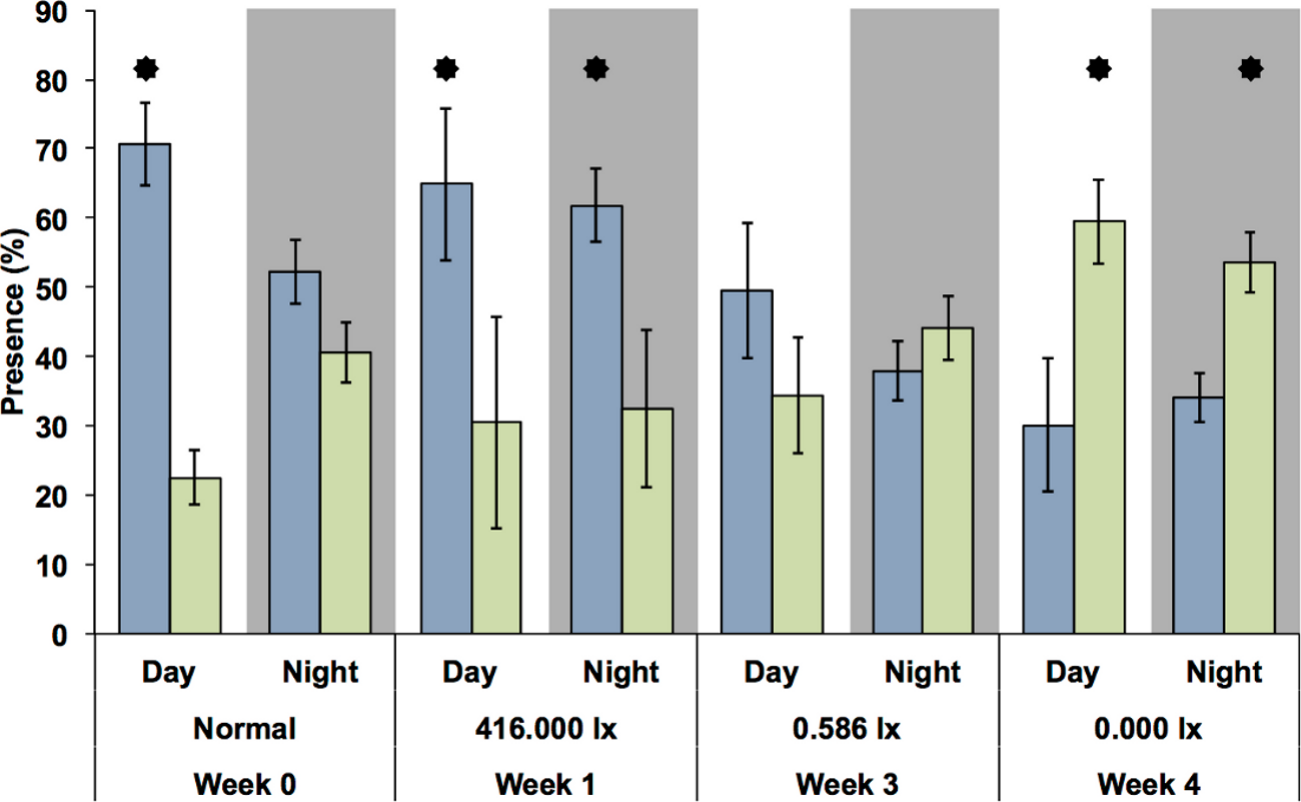
Presence (retention time) of *G. roeseli* in the upstream (blue) and downstream (green) chambers over the course of the experiment. Mean (± SE) presence (retention time) values are shown. The numbers 416, 0.586, and 0.0 lx are the light levels to which each treatment was exposed at night. “Normal” indicates no experimental night-time lighting was used. Asterisks indicate significance between upstream and downstream measuring chamber (Wilcoxon sign-rank test).

Because there was only one MFB device available, this experiment could only be carried out at the different light levels during different weeks and only with *G. roeseli*. Nevertheless, the time spent in the measuring chambers at the upper and lower end of the translucent tubes (Fig. 6) were similar to the results from the drift nets in the flumes. During the night, the time spent (presence) in the downstream chamber (i.e., indicating drift behavior) decreased with the onset of permanent light in the first week. However, the presence in the upstream chamber remained high, indicating considerable compensatory upstream activity. In week 3, presence in the downstream chamber increased for both night and day. By week 4, downstream activity was more frequent than upstream activity and was higher during the day than during the night.

### Light measurements in Berlin

The brightest light levels were recorded near a large billboard display on the river (Table 1), and resulted in light readings of approximately 2.5 lx at 20 cm and 1.4 lx at 40 cm under water. The highest light level at 50 cm was 0.4288 lx and was recorded where Friedrichstraße crosses the Spree River in the city center. The lowest light level recorded in the Berlin area was 0.0004 at the end of Ullsteinstr, where the street meets a canal of the Spree River in southern Berlin. This is in a locally green area with little development and where the only buildings are mostly garden homes that are primarily used in the summer. The measurements are all approximate because the turbidity of the water was extremely variable even from second to second. All measurements were taken with the light sensor pointing straight upward; light levels were noted to increase greatly if the sensor was angled toward the nearest light source.

**Table 1:** Light levels from streams in the Berlin metropolitan area. Light levels measured^a^ at various water bodies in the Berlin area at 50 cm depth, and in the flumes at 20 cm depth, with moonlight light levels for comparison.

Location/Flume/Moon	Light level (lx)
*Flume 8*	*0.0000*
Ullsteinstraße	0.0000
Alt-Gatow	0.0395
Stralauer Allee	0.0403
*Flume 7*	*0.0420*
Borsigturm	0.0434
Seestraße	0.0434
Großer Spreering	0.0537
Schäfersee	0.0614
*Flume 6*	*0.1040*
Hauptbahnhof	0.1123
*Flume 5*	*0.2800*
Full moon, clear sky, temperate latitude	0.3
Müggelseedamm^b^	0.3183
Friedrichstraße	0.4288
*Flume 4*	*0.5860*
*Flume 3*	*1.3100*
Kupfergraben	1.4000^c^
*Flume 2*	*4.1850*
*Flume 1*	*416.000*

**Notes.**

a All readings were taken with an ILT1700 light meter with the light sensor held vertical.

b This measurement was taken directly below a light on a bridge.

c This measurement was taken at a water depth of 40 cm; a measurement at 20 cm in this same location was 2.500 lx.

## Discussion

Surprisingly, we did not find evidence that gammarid drift was inhibited by artificial light at night. On the other hand, nocturnal drift increased over time, as hypothesized; however, the day drift showed an even higher increase, indicating that the increase might not have been due to acclimation to the light, but was rather a response to some other variable. Additionally, we found that drift rates and light levels were not linearly correlated. These results were reflected in the successive MFB experiments with *G. roeseli*. In week 1 the test organisms spent significantly more time in the upstream chamber, but by week 4 they spent significantly more time in the downstream than upstream chamber. These patterns emerged even though there was high variance in the MFB data. Highly variable activity as observed in the MFB experiments is a common and normal feature in gammarid behavior (Engelhardt, 2008) and is in accordance with the MFB results of Berghahn et al. (2012). The patterns in the MFB were the same for both *G. roeseli* and *G. pulex*, with relatively high activity levels in the dark at night treatment and decreasing activity levels in the most brightly lit treatment. This decrease in physical activity may be attributed to exhaustion driven by light stress.

Given previous studies have found a close relationship between exposure to light and decreased numbers of drifting macroinvertebrates (Anderson, 1966; Bishop, 1969; Brewin & Ormerod, 1994), we were surprised to find increased drift rates in all flumes with the onset of the light gradient. However, the majority of previous studies have looked at invertebrate drift in the summer months and found lower rates of drift during the day than at night (Anderson, 1966; Bishop, 1969; Flecker, 1992; Brewin & Ormerod, 1994); reviewed in Svendsen, Quinn & Kolbe (2004). However, the few studies that have analyzed invertebrate drift patterns in fall and winter have found that diurnal drift is higher than nocturnal drift (Williams, 1990; Bogatov & Astakhov, 2011).

We propose a few possible explanations for the lack of an effect of light treatment on the gammarid drift rates. They include: unnatural invertebrate densities, changes in chemical parameters in the flumes, parasitism, food availability, a lack of drift-feeding predators, and seasonal patterns in drift rates. We address these possible additional effects and argue that seasonal changes in drift rates are the most likely explanation for our unexpected results.

The gammarid densities in this experiment were comparable to the lower end of the range previously found in field abundance during the winter (Welton, 1979; Mortensen, 1982; Crane, 1994; Ladewig, 2004; Duran, 2007) and therefore representative. Most physical–chemical parameters that could have had an effect on physical activity (e.g. oxygen) remained almost constant over time; only temperature decreased during the experiment. Host–parasite relationships may also increase and modify the behavior and drift rates in *G. pulex and G. roeseli* (Lagrue et al., 2007). But we did not observe any obvious indications for parasitism in the specimens tested and even if present, they can be assumed to have been evenly distributed because the flumes were stocked from the same sources. The physical activity levels of each test animal during the dark at night treatment indicated valid measurements as a result of the light regime rather than food deprivation. While gammarids of 6–9 mg do feed even at water temperatures lower than 5°C, the periphyton biomass results gave no indication of food deprivation, which could be a reason for increased drift (Hildebrand, 1974; Hinterleitner-Anderson, Hershey & Schuldt, 1992). In any case, individuals in the light at night trials would have experienced the same food availability. Predators were not present in the experimental streams which may have promoted the increase in day drift catches because a lack of visual predators has been shown to result in aperiodic drift (Brewin & Ormerod, 1994). In this case, not only visual but also olfactorial detection of potential predators by infochemicals has to be taken into account (Brönmark & Hansson, 2012). Friberg et al. (1994) found, however, that *G. pulex* in Danish spring brooks had a preference for drifting at night, regardless of whether drift-feeding fish were present or not.

Finally, it is possible that the patterns in day and night drift were driven by seasonal changes. Bogatov & Astakhov (2011) saw increases in day relative to night drift during the winter in a far-northern Russian river. The increase in day drift coincided with the icing over of the river, leading to lower light levels in the water. Obviously, the water in our study did not freeze over. A previous study (Williams, 1990) also found a decrease in nighttime drift when temperatures decreased to 7.9°C while daytime drift and temperature were not related. This is consistent with our results and suggests that diel drift patterns may be subdued during the winter. Because we only took two samples (16 h night and 8 h day) in each 24 h sampling period, it is not possible to determine when the peak drift densities occurred. If the peak drift density changed to midday as temperatures dropped, that would support a dramatic seasonal change in behavior. On the other hand, if peak drift densities took place just before sunset, then it would not represent as much of a behavioral change.

### Future work

Research is needed to clarify whether gammarids and other invertebrates exhibit seasonal changes in diel drift patterns. Experiments to test explicitly the role of temperature changes and day length and night light levels in altering patterns of day and night drift will be especially helpful. Furthermore, we recommend taking hourly drift samples throughout experiments to further clarify when peaks in the drift occur in the winter.
